# Genes, age, and alcoholism: analysis of GAW14 data

**DOI:** 10.1186/1471-2156-6-S1-S119

**Published:** 2005-12-30

**Authors:** Victor Apprey, Joseph Afful, Jules P Harrell, Robert E Taylor, George E Bonney

**Affiliations:** 1 National Human Genome Center, Howard University, Washington DC 20059-0001 USA; 2 Department of Community Health and Family Medicine, Howard University, Washington DC 20059-0001 USA; 3 Department of Psychology, Howard University, Washington DC 20059-0001 USA; 4 Division of Medical Genetics, Department of Pediatrics, Howard University, Washington DC 20059-0001 USA; 5 Howard University Cancer Center, Howard University, Washington DC 20059-0001 USA; 6 Alcohol Research Center, Department of Pharmacology, Howard University, Washington DC 20059-0001 USA

## Abstract

A genetic analysis of age of onset of alcoholism was performed on the Collaborative Study on the Genetics of Alcoholism data released for Genetic Analysis Workshop 14. Our study illustrates an application of the log-normal age of onset model in our software Genetic Epidemiology Models (GEMs). The phenotype ALDX1 of alcoholism was studied. The analysis strategy was to first find the markers of the Affymetrix SNP dataset with significant association with age of onset, and then to perform linkage analysis on them. ALDX1 revealed strong evidence of linkage for marker *tsc0041591 *on chromosome 2 and suggestive linkage for marker *tsc0894042 *on chromosome 3. The largest separation in mean ages of onset of ALDX1 was 19.76 and 24.41 between male smokers who are carriers of the risk allele of *tsc0041591 *and the non-carriers, respectively. Hence, male smokers who are carriers of marker *tsc0041591 *on chromosome 2 have an average onset of ALDX1 almost 5 years earlier than non-carriers.

## Background

Past Collaborative Study on the Genetics of Alcoholism (COGA) results suggest that genetic linkage of alcohol dependence to markers on chromosomes 1, 2, 4, and 7 deserves further studies [1-3]. For Genetic Analysis Workshop 14 (GAW14), an expanded dataset with far denser marker sets has been provided. Our intent is to illustrate some of the models implemented in our software Genetic Epidemiology Models (GEMs) [4], in particular the regressive models for combined segregation and linkage analysis [5]. Previous analyses of COGA data have used regressive types of models as well. The analysis by Tiwari et al. provided strong association for chromosome 2 but no strong evidence of linkage [2], while the version by George et al. provided strong association for chromosomes 2 and 7 and nominal significance of linkage to chromosomes 1 and 4 [3]. Here we concentrate on age of onset of ALDX1 on a log-normal scale.

## Methods

We focused on ALDX1 and its age of onset. We used the given definition of alcoholism, ALDX1: classes 1, 2, 3, 4 were coded as unaffected and class 5 as affected. The data had the following characteristics: 143 pedigrees; 1,614 persons; 643 affecteds; 735 unaffected; 356 male smokers; 297 male non-smokers; 245 female smokers; and 470 female non-smokers.

We adapted the well known Elston-Stewart [6] likelihood framework for the analysis. The components of the likelihood model are the following: the population disease gene and marker frequencies, transmission probabilities, which for linkage analysis depend on the recombination fraction, *θ*, and the probability model for the penetrance function. The age-of-onset model of Elston [7] uses a mixture formulation for the penetrance function. Here, we use the simpler log-normal density model more common in survival analysis in which the unaffected are assumed censored at the age at last examination. The mean of the log-normal is a function of genotype and other explanatory variables, but the variance is constant across genotypes. This version of the log-normal age-of-onset model is implemented in our software GEMs, which makes it easy to specify interaction terms, add residual covariation as in the regressive models of Bonney [8,9], and Bonney et al. [5], and ascertainment corrections as in Bonney [10], as needed.

The mean age of onset on the log scale is a linear function of the disease genotype (assuming single locus and coded as a dummy variable), marker locus, sex, and smoking, also coded as dummy variables. Products of these variables are included to study interactions. The versions of the model applied to ALDX1 differed only in the specification of the mean age of onset, *μ*. Using the subscript *g *to denote genotype at the unobserved disease locus coded by the dummy variable *X*_*g*_, and subscript *m *for the marker locus, we applied one model for association and three different models for linkage analysis.

### Association model

*μ *= *β*_0 _+ *β*_*m*_•*X*_*m*_

Disease to marker association is tested by comparing the hypotheses *β*_*m *_= *0 *and *β*_*m*_*≠ 0 *using the likelihood ratio and its asymptotic chi-square with 1 degree of freedom. The analysis strategy first used marker association tests as a preliminary screening procedure to pick up markers for linkage study. Markers found significant at the 0.0001 level were tested further for linkage.

### Linkage model I

The mean age of onset depends on the interaction of marker and disease genotype:

*μ *= *β*_0 _+ *β*_*g*_•*X*_*g*_•*X*_*m*_.

### Linkage model II

The mean age of onset depends on the interaction of marker and disease genotype, and a linear sex effect:

*μ *= *β*_0_* + β*_*g*_•*X*_*g*_•*X*_*m *_+ *β*_*sex*_•*X*_*sex*_.

### Model III

The mean age of onset depends on the interaction of marker and disease genotypes, sex, smoking, and the, interaction of gender and smoking, thus

*μ *= *β*_0 _+ *β*_*g*_•*X*_*g*_•*X*_*m *_+ *β*_*sex*_•*X*_*sex *_+ *β*_*smoker*_•*X*_*smoker *_+ *β*_*sex_smoking*_•*X*_*sex*_•*X*_*smoking*_.

Note that the association model does not include the unobserved disease genotype, but the linkage models do. Note also that the linkage models include disease locus and marker locus interaction but not their "main effects". Doing so led to horrible convergence problems due to "over fitting". For a test of linkage, LOD scores were calculated as usual from the formula

LOD score = Log_10_{max likelihood (*0 *≤ *θ *≤ *1/2*)}/{max likelihood (*θ *= *1/2*)}.

The significance of the effects of covariates, sex and smoking, were tested by usual comparison of the estimates of the coefficients with their standard errors, making the justified assumption that the sample was large enough for the likelihood estimates to have approximate normal distribution.

## Results

Association and linkage results for observed marker genotypes are presented in Table 1. The markers presented are those that showed significant association with the age of onset of ALDX1. The association results identified five markers for ALDX1; these were then tested for linkage. For Model II, sex as a covariate is significant at *p*-value of 0.05 level. Females have a lower mean age of onset than males. The first (Model I) and second (Model II) linkage results revealed significant LOD scores for marker *tsc0041591 *on chromosome 2 and suggestive linkage for marker *tsc0894042 *on chromosome 3.

For Model III, using chromosome 2 marker *tsc0041591*, with the addition of smoking status and interaction of sex and smoking, the estimated regression coefficient for the interaction of sex and smoking was significant at the 0.05 level of significance. Male smokers have a lower mean age of onset than females. The linkage tests showed higher LOD scores for marker *tsc0041591 *on chromosome 2.

The LOD scores for chromosome 3 marker *tsc0894042 *are similar for Models I, II, and III. However, sex, smoking and their interaction are not significant at the 0.05 level. Hence the effect of chromosome 3 marker *tsc0894042 *on alcoholism appears to be purely genetic, while that of chromosome 2 marker *tsc0041591 *is significant by itself but is even more marked among smokers. Table 2 displays the mean ages of onset of ALDX1 according to sex, smoking history, and marker carrier status for allele 1, the risk allele of *tsc0041591 *on chromosome 2. The mean age of onset of all ALDX1 cases is 22.18 for males and 23.18 for females. The mean age of onset of the risk allele carriers is lower, 20.39 for males and 22.78 for females; and that of non-carriers is higher, 23.63 for males and 24.41 for females. The lowest mean age of onset of ALDX1 occurs in smokers who are risk allele carriers; their onset of ALDX1 occurs almost 5 years younger on average.

**Figure 1:**
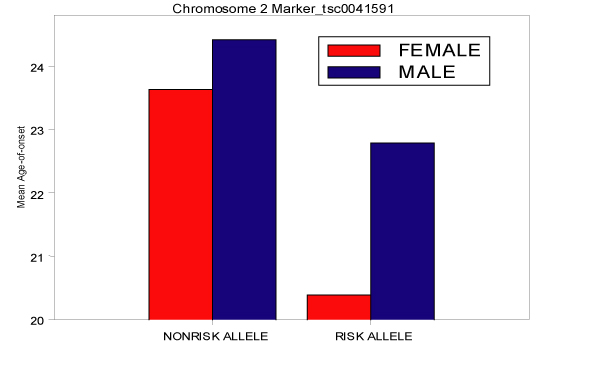
Age of onset by allele and sex.

**Figure 2:**
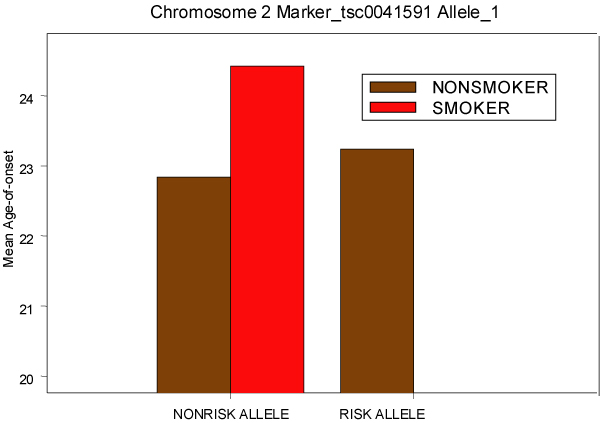
Age of onset by allele and smoking status.

Furthermore, Figure 1 shows that the difference in mean age of onset between males and females is significant for risk allele carriers but not significant for non-risk allele carriers. In Figure 2, the difference in mean age-of-onset for non-risk allele carriers for smokers and non-smokers is not significant while the difference for risk allele carriers is dramatically different.

## Discussion

Some general patterns are evident for Models I, II, and III. The markers that showed significant LOD scores without smoking as a covariate revealed higher LOD scores with smoking. The markers with non-significant LOD scores without smoking status as a covariate were also non-significant when smoking was added to the covariates.

We reduced the number of markers and chromosomes by performing association tests and then determining the evidence of linkage on the selected markers. However, in performing this reduction we are assuming no association leads to no evidence of linkage.

We selected markers that showed association at 0.01 significance level and tested them for linkage. There were many more markers significant at *p*-values of 0.05 for the association test. It is possible that we have missed some important markers in our conservative strategy of analysis.

## Conclusion

In summary, the markers *tsc0041591 *and *tsc0512083 *on chromosome 2 and *tsc0894042 *on chromosome 3 revealed strong or suggestive linkage for alcoholism. LOD score values increased among smokers for markers *tsc0512083 *and *tsc0512083 *on chromosome 2 but not for marker *tsc0894042 *on chromosome 3. The effect of sex on the genetics of alcoholism was not as strong as that of smoking.

Concerning our tool, GEMs, we found the speed of computation to be very slow for the genome scan data. The version used did not utilize parallel processing. Moreover, the largest LOD scores sometimes only reflected local optimum conditions. The solution is to use several different initial estimates. This has now been automatically implemented in GEMs.

## Abbreviations

COGA: Collaborative Study on the Genetics of Alcoholism

GAW14: Genetic Analysis Workshop 14

GEM: Genetic Epidemiology Models

## Authors' contributions

VA drafted the manuscript and performed statistical analyses. JA participated in the acquisition of data and analysis of data. JPH participated in the analyses and interpretation. RET participated in the data analysis and interpretation of results. GEB conceived of the study and help to draft the manuscript. All authors read and approved the final manuscript.

## Figures and Tables

**Table 1:** Linkage LOD scores for age of onset of ALDX1.

Chrom	Markers	Distance (cM)	LOD scores		
			
			Model I	Model II	Model III
1	*tsc0056724*	139.838	0.78	0.24	0.77
					
2	*tsc0041591*	200.911	3.25	3.39	3.75
	*tsc0512083*	200.914	0.99	1.05	1.32
					
3	*tsc0894042*	120.770	1.94	1.94	2.01
					
5	*tsc1640504*	96.473	0.66	0.65	0.63
					
10	*tsc0597718*	120.955	0.86	0.86	0.84

**Table 2:** Mean age of onset for ALDX1 and marker *tsc0041591 *allele 1.

		Allele 1 carriers (SEM)	All ALDX1 cases (SEM)	Non-carrier cases (SEM)
Sex	Male	20.39 (0.70)^a^	22.18 (0.40)	23.63 (0.65)
	Female	22.78 (0.95)	23.51 (0.60)	24.41 (1.12)
				
Smoker	Yes	19.76 (0.59)^a^	22.60 (0.43)	24.42 (0.75)
	No	23.24 (1.04)	22.50 (0.53)	22.83 (0.84)

^a^Significant at *p*-value of 0.05
